# The Glomerular Filtration Rate: From the Diagnosis of Kidney Function to a Public Health Tool

**DOI:** 10.3389/fmed.2021.769335

**Published:** 2021-11-25

**Authors:** Ana Maria Cusumano, Carmen Tzanno-Martins, Guillermo Javier Rosa-Diez

**Affiliations:** ^1^Centro de Educación Médica e Investigaciones Clínicas Norberto Quirno (CEMIC), Buenos Aires, Argentina; ^2^Independent Researcher, São Paulo, Brazil; ^3^Hospital Italiano de Buenos Aires, Buenos Aires, Argentina

**Keywords:** glomerular filtration rate, chronic kidney disease, MDRD study equation, CKD-EPI equation, cystatin C, creatinine clearance

## Abstract

The prevalence of chronic kidney disease (CKD) continues to increase worldwide, as well as the associated morbidity and mortality and the consequences on the patients' quality of life and countries' economies. CKD often evolves without being recognized by patients and physicians, although the diagnosis is based on two simple laboratory data: the estimated glomerular filtration rate (eGFR) and urine analysis. To measure GFR, the knowledge about the physiologic processes at the nephron level, the concept of clearance, and the identification of creatinine as a suitable endogenous marker for measuring the creatinine clearance (CrCl) had to be previously developed. On those bases, different equations to calculate CrCl (Cockcroft and Gault, 1976), or estimated GFR (four variables MDRD, 1999; CKD-Epi, 2009, among others) were generated. They all include creatinine and some demographic data, such as sex and age. However, to compare results throughout life or among laboratories, the creatinine determination must be standardized. In addition, the accuracy of these equations remains controversial in certain subgroups of patients. For these reasons, other mathematical models to improve CrCl estimation have been developed, such as when urine cannot be collected, in debilitated elderly patients and patients with trauma, diabetes, or obesity. Currently, eGFR in adults can be measured and reported immediately, using isotope dilution mass spectrometry traceable creatinine-based equations. In conclusion, based on knowledge obtained from renal physiology, eGFR can be used in the clinic for the diagnosis and early treatment of CKD, as well as a public instrument to estimate the prevalence.

## Introduction

The prevalence of chronic kidney disease (CKD) continues to increase worldwide, as well as the associated morbidity and mortality and the consequences on patients' quality of life and countries' economies. In the year 2018, a joint document of the ASN, ERA-EDTA, and ISN societies estimated that over 850 million people worldwide (11% of the total population) lived with kidney disease, about twice the number of diabetic patients estimated by the IDF (422 million) (1–3). An analysis of the Global Burden of Disease Study stated that CKD as cause of death rose from position 25 in the year 1990 to the 17th in 2015 (4). Another publication from the same study estimated that CKD as a mortality cause would ascend to the 5th place by 2040 (5). Besides, CKD is an independent risk factor for cardiovascular disease, and a risk multiplier in other non-communicable chronic diseases such as hypertension, diabetes, and cardiovascular (6). These data confirm that CKD is a major public health problem.

Any clinical situation resulting from a reduction in the number of functioning nephrons can evolve to CKD, defined by KDIGO guidelines as “abnormalities in kidney structure or function, present for 3 months, with implications for health.” The same guideline classifies CKD based on the cause, glomerular filtration rate (GFR) category, and albuminuria (7).

Arterial hypertension, diabetes, obesity, proteinuric nephropathies, race, family history, genetic diseases, low birth weight, aging, among others, are risk factors for the CKD (8, 9). Early detection and treatment of potentially reversible risk factors and CKD allow to delay progression and its associated complications as well as reduce the risk of cardiovascular disease (10, 11).

In the real world, CKD is a silent disease that often evolves unrecognized by the patients and physicians, although the diagnosis is based on the two simple laboratory data: the estimated GFR (eGFR) and urine analysis (screening for albuminuria/proteinuria) (10). Early diagnosis of CKD by the general practitioners and generalists would contribute to retard progression, and reduce morbidity and mortality associated to CKD and its associated risk factors (12).

Glomerular filtration rate continues to be the best global index of kidney function, both in health and in disease, as it represents the excretory capacity of the kidney, correlates directly with the kidney functioning mass, to classify CKD in stages according to the risk of progression, and to calculate the drug dosing and preparing for the invasive studies. xx

Early diagnosis of CKD by the general practitioners and generalists would contribute to retard progression and reduce associated morbidity and mortality. Albuminuria, an important predictor of CKD progression, will not be analyzed in this article. Therefore, the evaluation of kidney function and the presence or absence of proteinuria/albuminuria should be part of any routine health evaluation, and desirable when conducting population health surveys.

The present manuscript, after a brief historical description on the milestones that paved the way since the emerging physiological concepts of filtration, reabsorption, and excretion at the nephron, will focus on the present concepts of eGFR, and how it can be applied in the clinic and as a public health tool. Finally, different eGFR equations derived from creatinine and cystatin C and demographic data used for the diagnosis in patients and as a public health instrument will be described.

### The Identification of the Process of Glomerular Filtration to Measuring GFR in the Clinic

Knowledge of kidney physiology began in the mid-19th century, when Carl Ludwig (1816–1895) developed the concept of glomerular filtration. In his thesis, he identified the glomerulus as a filter, where urine formation began; this filter was submitted to physical and chemical forces, driven by the hydrostatic pressure generated by the heart, and regulated by the contraction and vasodilatation of the afferent and efferent arterioles. He went further, speculating that the filtered volume decreased along the tubules due to reabsorption, in order to concentrate the final products at the urine (13, 14).

In 1874, Rudolf Heidenhain (1834–1897) injected a dye, indigo carmine, in hypotensive anuric rabbits; after 15 min he removed the kidneys and identified the dye in tubular cells. He deduced that secretion from blood into the tubule occurred that meant an active tubular transport mechanism (15).

Arthur Cushny (1866–1926) in 1917 reasoned that Ludwig's theory (the glomerulus as a filter) implied a large volume of water, and near all the filtered glucose, amino acids, sodium, and other solutes should be in the ultrafiltrate. And, as these solutes are present at different concentrations in the plasma, the reabsorption of glucose, amino acids, and others dissolved substances present in urine should happen according to their respective blood levels. He concluded that there was a threshold for differential reabsorption of some solutes (16).

In 1924, Alfred Richards (1876–1966) and Joseph Wearns (1893–1984) published their results for the filtration process, infusing epinephrine into the glomerulus of an anesthetized frog, and observing the hemodynamic effects on the afferent and efferent arterioles and the resulting ultrafiltrate. They confirmed that the free-protein ultrafiltrate was due to filtration at the glomerular tuft, the solutes were filtered and reabsorbed at the tubular level, and there was a threshold for glucose reabsorption, corroborating the differential reabsorption of filtered solutes in the tubule (17).

At this point, the mechanism of filtration, secretion, and reabsorption in the nephron had been proved, but to transfer the concept of GFR to the clinic, it was still necessary to find a solute removed only by filtration, and not reabsorbed or secreted in the tubule. In 1926, Paul Rehberg identified creatinine as that solute, as it was produced by the body itself, filtered and, presumably, it was not reabsorbed or excreted (18).

Donald Van Slyke (1883–1971), in 1928, introduced the concept of “clearance,” regarding urea, as the volume of blood that would be totally cleared of it in a minute when urine flow exceeded 2 ml/min. The clearance technique was quickly applied to different solutes and became itself a fundamental tool in kidney physiology (19). Applying this concept, in 1937, Homer Smith measured GFR using inulin, a substance he had proved previously was excreted exclusively by glomerular filtration. After that, for many years, inulin was the gold standard to measure true GFR (20).

The clearance concept was fundamental, not only for studying the formation of urine or kidney physiology, but because it provided a simple tool to be used in the clinic, as GFR could be measured as creatinine clearance (CrCl). Since then, over many years, CrCl has been used in the clinic to evaluate GFR.

### Creatinine and Creatinine Clearance as Estimated GFR

Creatinine is a waste product of muscle metabolism, generated relatively constantly. It is almost eliminated by the glomerular filtration as it is a small molecule (113 Daltons) not bound to proteins. However, as its concentration depends on muscle mass, it is different in men and women, and may change according to the protein diet and muscle mass (21).

In 1886, Max Jaffe (1841–1911) noticed that creatinine in contact with picric acid in an alkaline solution developed an orange-red color, proportional to creatinine concentration (22). Years later, in 1914, based on the Jaffe's experiment, Otto Folin measured the creatinine in deproteinized blood, incorporating Jaffe's reaction to the clinical diagnosis (23). By this time, creatinine had been identified as a substance removed only by filtration, the concept of clearance was introduced and widely applied in his studies by Homer Smith, and the determination of serum creatinine was available. All the conditions were met for using CrCl as a proxy for GFR in clinical practice.

At present, creatinine is one of the most frequent laboratory determinations: easy to perform, available almost everywhere, and cheap. It can be determined by enzymatic or colorimetric methods. However, as every analyte, serum creatinine measure is exposed to random error (performed by the operator) and systematic error (depending on the material, the instrument, and the process). Standardizing its determination as a method with traceable calibration to isotope dilution mass spectrometry (IDMS) reduces biases, improving the accuracy of creatinine determination (24, 25).

Isolated creatinine is not a good marker to evaluate kidney function, as it increases when GFR is around 50 ml/min or below. Creatinine clearance is better to estimate GFR, but it has some disadvantages when evaluating kidney function. Creatinine is not excreted only by glomerular filtration, as a small fraction is secreted at the tubular level. This fraction increases as kidney function decreases and cannot be calculated individually (21). Also, in advanced CKD, the intestinal microbiota contributes to degrading creatinine, and this proportion also cannot be estimated (26). Therefore, when CKD is present, CrCl tends to overestimate GFR, and the difference increases as kidney function decline. This fact moved to search mathematical formulas based on creatinine and demographic factors, such as age, sex, body mass index, and race, to estimate GFR (eGFR).

Despite the limitations described, creatinine continues to be the most frequent marker used in the clinic to assess the function of kidney.

In some situations, a 24-h urine collection is mandatory, and the measured creatinine clearance is preferred in some patient groups to avoid misinterpretations. This is the case of a very low protein intake, such as vegetarians, high protein intake, creatine supplementation, diet rich in meat, some muscle mass abnormalities (malnutrition, amputation, and loss of muscle mass), rapid change in kidney function, before starting dialysis or in children and pregnant women (27, 28).

### The Development of Equations, Based on Creatinine, for Measuring eGFR

Equations to estimate GFR are widely used in day-to-day practice. More than 70 have been developed. In this publication, the most used and recent will be detailed.

One of the first ones was the Cockcroft–Gault (C–G) equation, available since the mid-1970s, that (or which) includes creatinine, sex, and weight, and is not adjusted for the body surface area (Table 1) (29). However, this equation correlates more to CrCl than to GFR. Besides that, the creatinine method used in the development of the C–G equation is no longer in use, and samples from the study are not available to compare the results to standardized creatinine values (30). Anyway, this equation has been and continues to be widely utilized, in part because many pharmacokinetic studies had been performed in the previously used C–G equation, before the standardization of serum creatinine traceable to IDMS (31, 32).

**Table 1:** Most used creatinine and cystatin C equations to estimate glomerular filtration rate (eGFR).

**Cockcroft-Gault equation**
Creatinine Clearance = $\frac{140-\text{age }\left(\text{years}\right)\text{ x weight }\left(\text{kg}\right)}{72\text{ x serum creatinine }\left(\text{mg}/\text{dl}\right)}\text{ x }0.85\;\left(\text{if female}\right)$
**MDRD-4 (simplified)** Estimated Glomerular Filtration Rate (mL/min/1.73 m^2^) == 175 (Serum Creatinine in mg/dl × 0.011312)^−1.154^ × (age in years )^−0.203^ × (0.742 if female) × (1.212 if African American/black)
**CKD-EPI (2009)** Estimated GFR = 141 x min(S_Cr_/κ, 1)^α^ x max(S_Cr_ /κ, 1)^−1.209^ x 0.993^Age^ x 1.018 [if female] x 1.159 [if Black] SCr (standardized serum creatinine) = mg/dL., K = 0.7 (females) or 0.9 (males), α = −1.329 (female) or−0.411 (male), Min = indicates the minimum of S_Cr_/K or 1, max = indicates the maximum of S_Cr_/K or 1, Age = Years
**FAS (2016)**1) Estimated GFR = 107.3/(SCr/ *Q*) for age ≤ 2 to ≤ 40 years 2) Estimated GFR = 107.3/(Scr/ *Q*) x 0.988 ^(age−40)^ for age > 40 years *Q*: the mean or median SCr value for age/sex-specific healthy populations
**CKD-EPI cystatin C equation** Estimated Glomerular Filtration Rate (mL/min/1.73 m^2^) == 133 × min(Scys/0.8, 1)^−0.499^ × max (Scys/0.8, 1)^−1.328^ × 0.996^Age^ [ × 0.932 if female] Scys = serum cystatin C, min indicates the minimum of Scr/κ or 1, and max indicates the maximum of Scys/κ or 1
**CKD-EPI creatinine-cystatin C** Estimated Glomerular Filtration Rate (mL/min/1.73 m^2^) =135 × min(Scr/κ, 1)*^α^* × max(Scr/κ, 1)^−0.601^ × min(Scys/0.8, 1)^−0.375^ × max(Scys/0.8, 1)^−0.711^ ×0.995^Age^ [ ×0.969 if female] [ ×1.08 if black]Scr = serum creatinine, Scys = serum cystatin C, κ is 0.7 for females and 0.9 for males, α is −0.248 for females and −0.207 for males, min indicates the minimum of Scr/κ or 1, and max indicates the maximum of Scr/κ or 1.

Similar equations require other data like patient height and/or weight; many times this information is not recorded or correctly recorded, favoring erroneous results.

In 1999, Levey and associates developed seven equations applying a regression model to predict eGFR, using data of 1,628 patients enrolled in the baseline period of the Modification of Diet in Renal Disease (MDRD) study. The equation that gave the best agreement with iothalamate-measured GFR was the six variable equation, valid for a standard body surface of 1.73 m^2^ (33):

GFR = 170 × [PCr]-0.999 × [Age]-0.176 × [0.762 if women] × [1.180 if African American/black] × [SUN]-0.170 × [Alb]+0.318

In 2000, Levey and coworkers proposed the simplified four-variable MDRD equation that correlates very well with the six-parameter equation proposed before (Table 1) (34). This formula, originally defined for serum creatinine measured by the old Jaffe method, was re-expressed with the serum creatinine calibrated to an assay traceable to IDMS in 2006 (35). A recommendation to convert Jaffe-measured creatinine into reference method-based procedures (SCr_Jaffe ×0.95 @ SCr_enzyme) if the reference procedure used for the calibration was IDMS has been proposed but a full agreement for doing so has not arisen (25).

The MDRD GFR has little bias compared with measured GFR with urinary clearance of iothalamate under 60 ml/min/1.73 m^2^, but underestimated the measured GFR at higher levels (36). These results are expected, as any equation reflects the characteristics of the population from which it derives; and the MDRD study was performed on the population with CKD.

In the year 2009, an improved creatinine-based equation, the CKD–EPI Collaboration (Chronic Kidney Disease Epidemiology Collaboration) was published, based on new methods for measuring creatinine, valid for all stages of renal insufficiency (Table 1). To work out the CKD–EPI equation, the authors took data from 8,254 participants of 10 studies (development set), 3,896 subjects from 16 studies (validation set) and, 16,032 individuals from NHANES (National Health and Nutritional Survey) for prevalence. The new equation performed significantly better than the MDRD study equation, especially at higher GFR, with lesser bias, improved precision, and greater accuracy (37).

This equation is still the most accurate GFR estimating equation evaluated in large diverse populations, applicable for the general clinical use. It provides lower estimates of the prevalence of decreased eGFR, and is useful as a trial measure for decreased eGFR and to replace the MDRD Study equation for routine reporting of serum creatinine-based eGFR by clinical laboratories (38).

A systematic search of MEDLINE, between 1999 and 21 October 2011, was performed by Earley et al. to review the GFR estimating equations performance; 12 studies were selected. In those from North America, Europe, and Australia, the authors corroborated, once again, that the CKD–EPI equation performed better at higher GFRs (> 60 ml/min/1.73 m^2^) and the MDRD equation behaved better at lower GFRs (<60 ml/min/1.73 m^2^). In Asian or African populations, neither equation worked as well as in the other regions (39).

KDIGO CKD Guidelines recommend clinical laboratories to report eGFR in adults using the 2009 CKD–EPI equation (7), provided creatinine determination is traceable to IDMS.

The inclusion of race in the eGFR equation has been questioned, even though, in adults, age, sex, weight, height, and race are surrogates of muscle mass. In the original MDRD and CKD–EPI equations, the inclusion of race (Black/non-Black) improved accuracy (40). It has been argued that to exclude race from the eGFR equations would provoke a systematic under interpretation of measured GFR. To disclosure about the use of race when estimating GFR, or to accept denial to identify race from the patient, or to use a cystatin C confirmatory test has been proposed as a way to overcome the conflict (41, 42). In September 2021, the National Kidney Foundation and the American Society of Nephrology Joint Task Force on Reassessing the Inclusion of Race in Diagnosing Kidney Diseases recommended a new 2021 CKD-EPI creatinine eGFR equation which does not include race to estimate GFR (Table 1). They recommend for the United States immediate implementation of the CKD-EPI creatinine equation refit without the race variable in all laboratories, and to facilitate increased, routine and timely use of cystatin C, as combining creatinine and cystatin C is more accurate (43).

Finally, in 2016, Pottel et al. developed a novel equation, the full age spectrum (FAS) equation, to estimate the GFR across all over the age spectrum since available equations lack continuity with aging (the Schwartz equation for pediatrics, the CKD–EPI equation for adults under 70 years age, and the BIS-1 for older than 70 years old). This new equation is normalized on serum Cr (SCr/*Q*) for age (children and adolescents) and gender (adolescents and adults), being *Q* the median serum Cr from a specific healthy subpopulation. In the validation study, 6,870 healthy and kidney disease caucasian and from the non-African origin individuals, of whom 765 were children and adolescents <18 years old and 1,748 elderly higher than 70 years old, participated. For validation, measured GFR was performed using inulin or iothalamate or iohexol clearance (Table 1) (44). The FAS equation that can be used in ages <2–100 years old, resulted less biased and more accurate than the Schwartz equation for children and adolescents, and less biased and as accurate as the CKD–EPI equation for adults under or over 70 years old.

### The Development of Equations Using Cystatin C

Cystatin C was described for the first time in 1961 (45). As creatinine, it is an endogenous marker. It is a low molecular weight protein (13 kD) and consists of a chain of 120 amino acids. Produced constantly by all nucleated cells of the body, it filters freely through the glomerulus and is totally reabsorbed and catabolized by the proximal tubular cells. Muscle mass, age, sex, or diet do not affect its concentration; these characteristics make Cystatin C useful in groups with reduced muscle mass (46, 47).

To improve the accuracy of eGFR several equations have now been developed using either cystatin C alone or cystatin C in combination with creatinine. Cystatin C-based equations have advantages over the creatinine-based equation as they are less influenced by age, sex, and race (48). The 2012 CKD–EPI creatinine-cystatin C equation is more accurate than the 2009 CKD–EPI creatinine and 2012 CKD–EPI cystatin C equations and it is useful as a confirmatory test for decreased eGFR as determined by serum creatinine-based eGFR (Table 1) (49).

Despite its greater usefulness, cystatin C has not displaced creatinine for GFR estimation in clinical practice, possibly due to its higher cost and lower availability.

#### Clinical Situations Where mGFR Is Needed

In some clinical situations, such as patients with anorexia nervosa, cirrhosis, obesity, evaluation of living kidney donors, prescribing nephrotoxic drugs with a narrow therapeutic window, pharmacokinetic studies of drugs excreted by the kidney, or any situation in which eGFR is unreliable, it is reasonable to measure GFR (mGFR), despite the added cost and time and resources consuming (7, 50–52). At present, iothalamate or 51Cr-EDTA or 99Tcm-DTPA urinary clearance or 99Tcm-DTPA or iohexol plasma clearance are the accurate methods for determining mGFR (52–55). In patients with large edema or ascites, urinary clearance should be employed (55).

Iohexol, a low-cost non-toxic non-ionic contrast agent, has some advantages for the plasma clearance such as simplicity, low cost, stability, and low interlaboratory variation. Besides that, it is not radioactive, is excreted almost exclusively by the kidney, is neither secreted nor reabsorbed at the tubular level, has low protein binding, and correlates with inulin renal clearance (56, 57). It is contraindicated in patients with allergy to iodine.

## Conclusion

From the knowledge that emerges from renal physiology, laboratory medicine, epidemiology, and biostatistics, have emerged equations that constitute tools not only for the clinical care of patients, but also to establish the prevalence of CKD, and consequently implementing the public health policies aimed to reduce it.

The inclusion of the automatic calculation of GFR in the laboratory reports of creatinine determinations constitutes a useful tool for daily practice, and from public health perspectives for population screening for CKD.

CKD–EPI Study equation continues to be the most used in general practice and from public health perspective. Recently, the FAS equation emerged as a promising option to estimate eGFR for all ages (from 2 to 100 years old).

Further improvement in GFR estimating equations will require development in more broadly representative populations, such as diverse racial and ethnic groups, use of multiple filtration markers, and evaluation using statistical techniques to compare eGFR to mGFR.

## Author Contributions

All authors listed have made a substantial, direct, and intellectual contribution to the work and approved it for publication.

## Conflict of Interest

The authors declare that the research was conducted in the absence of any commercial or financial relationships that could be construed as a potential conflict of interest.

## Publisher's Note

All claims expressed in this article are solely those of the authors and do not necessarily represent those of their affiliated organizations, or those of the publisher, the editors and the reviewers. Any product that may be evaluated in this article, or claim that may be made by its manufacturer, is not guaranteed or endorsed by the publisher.
